# Telehealth effectiveness for pre‐exposure prophylaxis delivery in Brazilian public services: the Combine! Study

**DOI:** 10.1002/jia2.26173

**Published:** 2023-09-27

**Authors:** Alexandre Grangeiro, Lorruan Alves do Santos, Denize Lotufo Estevam, Rosemeire Munhoz, Érico Arruda, Renata Amaral de Moraes, Lisiane de Quadros Winkler, Lis Aparecida de Souza Neves, Juliane Cardoso Villela Santos, Mariele Kruppa, Eliana Miura Zucchi, Maria Mercedes Escuder, Andréa Fachel Leal, Mitti Ayako Hara Koyama, Maria Fernanda Tourinho Peres, Marcia Thereza Couto, José Eluf Neto, The Combine! Research Group

**Affiliations:** ^1^ Faculdade de Medicina Universidade de São Paulo São Paulo Brazil; ^2^ Centro de Referência e Treinamento DST/Aids São Paulo Brazil; ^3^ Hospital São José de Doenças Infecciosas Fortaleza Brazil; ^4^ Serviço de Atenção Especializada do Centro de Saúde IAPI Porto Alegre Brazil; ^5^ Prefeitura Municipal de Ribeirão Preto, Ribeirão Preto São Paulo Brazil; ^6^ Centro de Orientação e Aconselhamento Curitiba Brazil; ^7^ Programa de Pós‐Graduação em Saúde Coletiva Universidade Catolica de Santos Santos São Paulo Brazil; ^8^ Instituto de Saúde Secretaria de Estado da Saúde de São Paulo São Paulo Brazil; ^9^ Instituto de Filosofia e Ciências Humanas Universidade Federal do Rio Grande do Sul Porto Alegre Brazil; ^10^ Kamiyama Consultoria em Estatística Ltda São Paulo Brazil

**Keywords:** Brazil, effectiveness, human immunodeficiency virus, pre‐exposure prophylaxis, prevention, telehealth

## Abstract

**Introduction:**

Pre‐exposure prophylaxis (PrEP) delivery based on user needs can enhance PrEP access and impact. We examined whether telehealth for daily oral PrEP delivery could change the indicators of care related to prophylactic use in five Brazilian public HIV clinics (testing centres, outpatient clinics and infectious disease hospitals).

**Methods:**

Between July 2019 and December 2020, clients on PrEP for at least 6 months could transition to telehealth or stay with in‐person follow‐up. Clients were clinically monitored until June 2021. A desktop or mobile application was developed, comprising three asynchronous consultations and one annual in‐person consultation visit. Predictors influencing telehealth preference and care outcomes were examined. The analysis encompassed intent‐to‐treat (first choice) and adjustments for sexual practices, schooling, age, duration of PrEP use and PrEP status during the choice period.

**Results:**

Of 470 users, 52% chose telehealth, with the adjusted odds ratio (aOR) increasing over time for PrEP use (aOR for 25–months of use: 4.90; 95% CI: 1.32–18.25), having discontinued PrEP at the time of the choice (aOR: 2.91; 95% CI: 1.40–6.06) and having health insurance (aOR: 1.91; 95% CI: 1.24–2.94) and decreasing for those who reported higher‐risk behaviour (aOR for unprotected anal sex: 0.51; 95% CI: 0.29–0.88). After an average follow‐up period of 1.6 years (95% CI: 1.5–1.7), the risk of discontinuing PrEP (not having the medication for more than 90 days) was 34% lower with telehealth (adjusted hazard ratio: 0.66; 95% CI: 0.45–0.97). When adjusted by mixed linear regression, no differences in adherence (measured by mean medication possession rate) were found between in‐person and telehealth (*p* = 0.486) or at pre‐ and post‐telehealth follow‐ups (*p* = 0.245). Sexually transmitted infections increased between the pre‐follow‐up and post‐follow‐up choices and were not associated with in‐person or telehealth (*p* = 0.528). No HIV infections were observed.

**Conclusions:**

Our findings indicate that telehealth for PrEP delivery can enhance service rationalization and reinforce the prevention cascade. This approach reduces prophylaxis interruptions and is mainly preferred by individuals with lower demands for healthcare services.

## 1 INTRODUCTION

Although pre‐exposure prophylaxis (PrEP) is associated with a reduced HIV incidence [1, 2, 3, 4], its low access [5] and inconsistent use over time [6] have compromised its population‐level effectiveness. Telehealth has emerged as a promising strategy for minimizing difficulties related to aspects of service organization, clinical follow‐up requirements and the vulnerability of HIV‐affected populations [6, 7, 8] by allowing users more significant rationalization of services and autonomy [9, 10, 11, 12, 13, 14]. The feasibility [13, 15] and acceptance [10, 11, 16, 17] of PrEP delivery through telehealth are higher in high‐income countries but unknown in middle‐ and low‐income countries [18]. Robust evaluations of telehealth are thus necessary to increase evidence of the effectiveness of PrEP, including adherence and persistence, with more incredible benefits for settings where access and use are challenging.

Telehealth has been used in different ways for PrEP delivery and accelerated by the coronavirus disease 2019 (COVID‐19) outbreak [19]. While some allow all procedures to be performed at home with self‐collection of tests and medication delivery by mail [12, 13], others adopt hybrid approaches [9, 20, 21]. Synchronous consultations [9, 12] and asynchronous procedures [13] have also been evaluated.

The high acceptance [10, 11, 16] of telehealth is attributed to its agility, practicality and privacy [11, 16, 20]. Telehealth is also associated with greater diversification of the population profile on PrEP [12], more excellent geographic coverage [9] and an increase in the number of higher‐risk people on PrEP [16]. Additionally, telehealth for screening and follow‐up increases PrEP uptake [15] and persistence [9, 12]. However, most studies included small sample sizes and lacked comparison groups, making it challenging to understand whether these results were due to telehealth.

Therefore, we developed a telehealth protocol that allows PrEP clients to choose between asynchronous clinical follow‐up using a web platform and in‐person consultations. We evaluated the acceptability and predictive factors of choice for telehealth and the effect of telehealth follow‐up on discontinuation of PrEP, adherence and frequency of occurrence of sexually transmitted infections (STIs) and HIV infection.

## 2 METHODS

### 2.1 Combine! Study

The evaluation occurred as part of a demonstration study [22] of daily oral PrEP among cisgender and transgender people≥ 16 years of age, with a higher risk of HIV, in five cities of different regions of Brazil. Different service modalities were selected, including two outpatient clinics (Ribeirão Preto, Southeast and Porto Alegre/South), two testing centres (São Paulo, Southeast and Curitiba, South) and one infectious disease hospital (Fortaleza, Northeast). The context and characteristics of the services are outlined in Table 1. Since November 2016, the follow‐up to PrEP has included quarterly consultations, laboratory examinations and semi‐annual application of a behavioural questionnaire.

**Table 1:** Characteristics and context of participating services

	Municipal level		State level	
City/State/Region	Demographics^a^ (2021)	HIV epidemiology^b^	PrEP profile^b^	Service feature
Porto Alegre/Rio Grande do Sul/South	State's capital; population (millions): 1.4; HDI^c^: 0.805	Aids incidence (per 100,000 inhabitants): 47.2; Late diagnosis (CD4<350 cell/mm^3^): 41%; Retention antiretroviral treatment: 72%; HIV viral suppression: 75%	People on PrEP: 2938 (public system: 93%); Rate PrEP people/HIV‐positive people: 0.134; PrEP interruption (%): 32.6%	Basic Health Unit, PrEP inserted in the HIV clinic, test collection in the public network
Curitiba/Paraná/South	State's capital; population (millions): 2.0; HDI: 0.823	Aids incidence (per 100,000 inhabitants): 18.2; Late diagnosis (CD4<350 cell/mm^3^): 48%; Retention antiretroviral treatment: 74%; HIV viral suppression: 82%	People on PrEP: 2995 (public system: 85%); Rate PrEP people/HIV‐positive people: 0.227; PrEP interruption (%): 38.6%	Specialized HIV services, PrEP inserted in the testing centre, test collection in the service
São Paulo/São Paulo/Southeast	State's capital; population (millions): 12.4; HDI: 0.805	Aids incidence (per 100,000 inhabitants): 17.9; Late diagnosis (CD4<350 cell/mm^3^): 43%; Retention antiretroviral treatment: 73%; HIV viral suppression: 80%	People on PrEP: 23,598 (public system: 91%); Rate PrEP people/HIV‐positive people: 0.315; PrEP interruption (%): 41.5%	Specialized HIV service, PrEP inserted in a testing centre and STI clinic, test collection and laboratory in the service
Ribeirão Preto/São Paulo/Southeast	Country town; population (millions): 0.7; HDI: 0.800	Aids incidence (per 100,000 inhabitants): 14.0; Late diagnosis (CD4<350 cell/mm^3^): 35%; Retention antiretroviral treatment: 79%; HIV viral suppression: 80%	People on PrEP: 23,598 (public system: 91%); Rate PrEP people/HIV‐positive people: 0.315; PrEP interruption (%): 41.5%	Specialized HIV service, PrEP inserted in the outpatient clinic, collection of tests in the service
Fortaleza/Ceará/North East	State's capital; population (millions): 2.7; HDI: 0.735	Aids incidence (per 100,000 inhabitants): 18.4; Late diagnosis (CD4<350 cell/mm^3^): 58%; Retention antiretroviral treatment: 70%; HIV viral suppression: 82%	People on PrEP: 18,891 (public system: 94%); Rate PrEP people/HIV‐positive people: 0.120; PrEP interruption (%): 29.8%	Infectious disease hospital, PrEP inserted in the outpatient clinic and day hospital, collection and laboratory in the service
Brazil	Population (millions): 213.3; HDI: 0.754	Aids incidence (per 100,000 inhabitants): 16.5; Late diagnosis (CD4<350 cell/mm^3^): 28%; Retention antiretroviral treatment: 71%; HIV viral suppression: 79%	People on PrEP: 59,471 (public system: 89%); Rate PrEP people/HIV‐positive people: 0.071; PrEP interruption (%): 37.6%	

^a^ Source: Brazilian Institute of Geography and Statistics and UNDP‐Brazil.

^b^ Source: Brazilian Ministry of Health.

^c^ Human Development Index.

The study has been registered in the “Brazilian Registry of Clinical Trials” with “The Universal Trial Number”: U1111‐1165‐7889s.

### 2.2 Telehealth protocol

The telehealth protocol included three quarterly remote assessments and annual in‐person evaluations. A web platform, accessible via cell phone or computer, was developed in consultation with professionals and clients for the clinical evaluations (see Supplementary Material). A total of 10 days before the scheduled consultation date, clients completed an online clinical form on this platform, which included 17 questions related to signs and symptoms of acute HIV infection or STIs, adverse events, adherence, sexual practices, drug use and mental health issues. Additionally, they had to attach recent laboratory results (minimum: HIV, syphilis, hepatitis and creatinine) to the platform (performed in the last 7 days). These tests could be conducted at public or private laboratories or directly at the PrEP service provider (Combine! service). The clinical evaluations were performed asynchronously by a physician on the platform. Based on their discretion, the physician could opt for a new PrEP prescription online or schedule an in‐person consultation for clinical verification. Clients were able to schedule consultations at their convenience. Medication was dispensed at the service pharmacy. Suspected STIs did not prevent clients from accessing PrEP; however, timely clinical investigation of any suspicions was recommended. The platform also sent clients reminders, links to exam requisition forms and PrEP prescriptions. A chat tool was available for clients to communicate with the service and access their follow‐up history. Participants were instructed to seek assistance if they experienced symptoms of STIs, acute HIV infection or any other sexual health issues. Professionals underwent training to implement the telehealth system, while clients received instructions from a professional on how to use the platform and follow the telehealth protocol.

All the participants consented to participate in the study. This study was approved by the Ethics Committees of the Clinical Hospital of the School of Medicine of the University of São Paulo (3438329/2019) and the Pan American Health Organization (PAHO‐2019‐06‐0048).

### 2.3 Eligibility, telehealth delivery and observation period

Telehealth was offered between July 2019 and December 2020 to 740 individuals who had started PrEP at least 6 months earlier (period adopted to allow familiarization with the service and PrEP use). For those with regular PrEP use, telehealth was offered preferentially at consultation, and for those who discontinued use, contact was made by telephone, social networks or e‐mail. Clients can change the type of follow‐up at any time.

After the first evaluation, individuals without internet access (*n* = 5) or clinical conditions requiring in‐person evaluation (*n* = 7), defined at the physician's discretion, were excluded. Therefore, 728 PrEP users were eligible for telehealth.

The observation of the studied outcomes occurred from July 2019 to June 2021 and involved both telehealth and in‐person follow‐up with clients. Clients who discontinued PrEP, moved to another city, switched to the event‐driven regimen or contracted HIV were censored at the final observation date. Clients who had changed cities but still had the chance to undergo the selected follow‐up were included in the observation.

### 2.4 Acceptability, reasons for choice and predictive factors

Acceptability was analysed based on the first choice of follow‐up type, and a prediction model for this choice was defined a priori with four levels of information. At the most proximal level, we assume that the alternative would be influenced by the experience with PrEP and access to healthcare (time of use and continuation of PrEP at the time of choice, city or follow‐up service, private healthcare and HIV testing before PrEP). At intermediate levels, the influence would occur due to HIV risk behaviour (type of sexual partnership, condom use during anal intercourse, risk self‐assessment) and vulnerability (sex work, drug use before or during sex and lifetime intimate partner violence). Demographic characteristics (gender identity, age, self‐reported skin colour and education) were recorded at the most distal level.

Data refer to the 6 months preceding enrolment in the Combine! Study, which was obtained by the applied questionnaire. Sex work was defined as sexual relations for money, favours, presents or self‐identification as a sex worker. The question used to define risk self‐assessment was: “How do you evaluate your risk of becoming infected with HIV with your casual partners, assigning a score from 1 to 10?”. Individuals rated ≥ 8 were classified as having a “higher risk perception.” Intimate partner violence was defined as coercion, humiliation, physical aggression with or without weapons, and sex and drug use without consent.

Participants completed an online questionnaire regarding their reasons for choosing telehealth, considering various factors related to the individual, the service and clinical follow‐up.

### 2.5 Discontinuation and adherence

Discontinuation was defined as a delay of ≥ 90 days since the last withdrawal was supposed to have occurred. This was measured at the end of the study, assuming that the date of the previous medication withdrawal was the date of discontinuation.

Adherence was measured using medication possession rates (MPR). For each medication withdrawal period, the number of pills delivered (NP) was divided by the number of days between withdrawal dates (DtW). Leftover medication (LO) or days without medication (DN) at each interval were added to the subsequent interval. Thus, MPR_1,2…_ = NP_1.._/((DtW_2…_‐DtW_1…_)+(LO_1…_,if LO_1…_>0 or DN_1…_ if DN_1…_>0)). An MPR <1 was considered non‐adherence.

### 2.6 Frequency of STIs and HIV

The incidence of STIs was identified as new reports of indicative signs and symptoms in the genitals, anus and mouth in medical records, irrespective of treatment, clinical conduct or laboratory test results.

HIV infection was screened at each visit or upon medication withdrawal using a third‐generation rapid test.

### 2.7 Analysis

Descriptive analysis was used to study the reasons for choosing telehealth. Associations with individual characteristics were verified using the chi‐square test. When differences in the distributions were observed, the standardized adjusted residual (Zres) was used to identify excess or absence | 1.96 | of the frequency.

The predictors of opting for telehealth were analysed using logistic regression. The model included the variables from distal to proximal levels, as defined in the predictive model. Variables with a significance level of ≤ 0.10 remained in the model for the analysis of subsequent levels and, in the final model, only variables with a significance of ≤ 0.05.

Assuming telehealth does not impact PrEP outcomes (e.g. discontinuation, non‐adherence, and occurrence frequency of HIV and STIs), this study compared these outcomes before and after telehealth implementation and between telehealth and in‐person follow‐up. The intention‐to‐treat method was used for the analyses. A complementary analysis was conducted, reclassifying individuals by the last type of clinical follow‐up performed at the end of the follow‐up period to consider the change in the type of follow‐up after the first choice.

Survival analysis for discontinuation was performed using the Kaplan−Meier method. Survival curves for each follow‐up type were compared using the log‐rank and Tarone−Wares tests. Cox regression was used to adjust for the effect of the follow‐up type on PrEP discontinuation, and Schoenfeld residuals were used to verify the model's assumptions.

Mixed linear models were employed to calculate the mean MPR for both telehealth and in‐person consultations in the pre‐ and post‐implementation periods of telehealth. This calculation was adjusted for variables reflecting risk, vulnerability, PrEP status and demographic characteristics. The study participants were considered random, and the other variables were considered fixed effects. The rates for each type of follow‐up were compared, and the interaction between time and type of follow‐up was analysed.

We also investigated whether non‐adherence was associated with either telehealth or in‐person follow‐ups. We employed logistic regression with MPR <1 tablet/day as the outcome variable to examine this. The model was adjusted for variables reflecting risk, vulnerability, PrEP status and demographic characteristics.

A generalized estimating equation model was used to analyse the evolution of STI occurrence in the pre‐ and post‐telehealth onset periods. The study subjects were considered clustering factors, and a negative binomial marginal distribution was employed, considering the exposure time. Analysis was adjusted for risk variables, vulnerability, PrEP status and demographic characteristics.

The analyses were conducted using a confidence interval of 95% (95% CI) in the software packages SPSS 20.0 and STATA 17.

## 3 RESULTS

### 3.1 Acceptability, reasons for choice and predictive factors

A total of 470 individuals (64.6% of the 728 eligible individuals) chose to undergo clinical follow‐up. Non‐choice occurred in 79.3% (214) of the cases because of the inability to locate the participant or because the participant did not select the invitation (56, 20.7%). Participants who did not choose (270) differed (*p* <0.05) by not using PrEP at the time of telehealth delivery (66.7%), were sex workers (31.5%), aged 16–25 years old (28.1%), were transgender women (14.1%) and had incomplete primary education (14.1%).

More than half (245, 52.1%) chose telehealth, which varied between 92.9% and 15.8%, depending on client characteristics (Table 2).

**Table 2:** Characteristics of the participants according to the chosen type of PrEP follow‐up in Combine! Study

	Choice of type of PrEP follow‐up					
	Telehealth		In person		Total	*p*
Characteristics	*N*	%	*N*	%		
**Demography**						
Gender identity (*n*, 469)						0.638
Cisgender man^a^	228	52.4	207	47.6	435	
Transgender woman	10	52.6	9	47.4	19	
Other	6	40.0	9	60.0	15	
Age range (years) (*n*, 469)						0.385
16–25	44	46.8	50	53.2	94	
26–45	179	54.1	152	45.9	331	
46 or more	21	47.7	23	52.3	44	
Self‐reported skin colour (*n*, 469)						0.585
Black	15	50.0	15	50.0	30	
Brown	60	48.0	65	52.0	125	
White	161	54.4	135	45.6	296	
Other	8	44.4	10	55.6	18	
Education (*n*, 469)						0.094
Incomplete elementary and middle school	9	36.0	16	64.0	25	
Complete high school and incomplete university	77	48.4	82	51.6	159	
Complete university	158	55.4	127	44.6	285	
**Vulnerability and risk to HIV (last 6 months)**						
Sex work (*n*, 470)						0.033
No	211	54.4	177	45.6	388	
Yes	34	41.5	48	58.5	82	
Sexual partnership (*n*, 467)						0.036
Only steady	21	58.3	15	41.7	36	
Only casual	97	45.5	116	54.5	213	
Steady and casual	125	57.3	93	42.7	218	
Active anal sex without a condom with a casual partner (*n*, 470)						0.003
No	124	54.6	103	45.4	227	
Up to half of the relations	93	57.1	70	42.9	163	
More than half of the relations	28	35.0	52	65.0	80	
Passive anal sex without a condom with a casual partner (*n*, 470)						0.224
No	148	55.4	119	44.6	267	
Up to half of the relations	54	46.2	63	53.8	117	
More than half of the relations	43	50.0	43	50.0	86	
Drug use before/during sexual encounters with more than half of the casual partners (*n*, 470)						0.914
No	206	52.0	190	48.0	396	
Yes	39	52.7	35	47.3	74	
Lifetime intimate partner violence in life (*n*, 470)						0.116
No	177	54.6	147	45.4	324	
1–3	63	48.5	67	51.5	130	
4–6	5	31.3	11	68.8	16	
Perception of increased risk with sexual						
partners (score 0–10) (*n*, 470)						
No (score from 0 to 7)	186	54.9	153	45.1	339	
Yes (score 8–10)	59	45.0	72	55.0	131	
**Experience with PrEP**						
Time of use of PrEP at the time of choice of follow‐up (months) (*n*, 470)						0.005
Up to 12	3	15.8	16	84.2	19	
13–24	104	54.5	87	45.5	191	
25–32	138	53.1	122	46.9	260	
Being on PrEP at the time of the choice of follow‐up (*n*, 470)						0.015
Discontinued	15	31.3	33	68.8	48	
In use	210	49.8	212	50.2	422	
City of follow‐up (*n*, 470)						<0.001
Curitiba (testing centre)	26	92.9	2	7.1	28	
Fortaleza (infectious diseases hospital)	25	44.6	31	55.4	56	
Porto Alegre (HIV outpatient clinic)	39	69.6	17	30.4	56	
São Paulo (testing centre)	144	46.8	164	53.2	308	
Ribeirão Preto (HIV outpatient clinic)	11	50.0	11	50.0	22	
**Access to healthcare**						
Has private healthcare insurance (*n*, 470)						0.001
No	90	43.7	116	56.3	206	
Yes	155	58.7	109	41.3	264	
HIV testing before PrEP uptake (*n*, 470)						0.442
No	22	46.8	25	53.2	47	
Yes	223	52.7	200	47.3	423	

^a^ All reported having sexual practices with men.

The reasons for choosing telehealth were primarily driven by individual factors rather than service‐related factors, as shown in Table 3. Two noteworthy factors were the belief that clinical visits were not essential for PrEP use (62.6%) and the convenience offered by telehealth (46.7%), such as avoiding absence from work and having flexibility in scheduling clinical evaluations. This pattern was consistent across the analysed characteristics. However, clients who were less socially vulnerable (i.e. men with a Zres of 3.2 and those with healthcare insurance with a Zres of 2.8) were more likely to consider clinical consultations unnecessary. Additionally, individuals with lower education (a Zres of 2.5) expressed concerns about experiencing stigma within healthcare services.

**Table 3:** Frequency of the reasons for opting for telehealth

Characteristic	PrEP does not require consultations in the services		User convenience		Negative quality of service		Reduce service demand		Anticipation of stigma	
	%	*p*	%	*p*	%	*p*	%	*p*	%	*p*
**Total (*n*, 244)**	62.6		46.7		29.7		27.2		5.7	
Gender identity		0.006		0.398		0.258		0.179		0.137
Cisgender man^a^ (*n*, 227)	64.8^b^		42.1		35.2		26.4		9.0	
Transgender woman (*n*, 10)	20.0^c^		50.0		10.0		10.0		10.0	
Other (*n*, 6)	33.3		16.7		33.3				33.3	
Skin colour		0.650		0.286		0.754		0.131		0.760
Black (*n*, 15)	46.7		60.0		26.7		13.3		6.7	
Brown (*n*, 58)	63.8		38.3		30.0		17.2		16.7	
White (*n*, 162)	63.0		42.4		36.4		29.6		6.7	
Other (*n*, 8)	62.5		22.2		33.3		12.5		22.2	
Education		0.022		0.580		0.190		0.056		0.009
Incomplete elementary and middle school (*n*, 9)	22.2^c^		33.3		11.1		··		33.3^b^	
Complete high school and incomplete university education (*n*, 77)	58.4		38.0		30.4		19.5		3.8^c^	
Complete university (*n*, 157)	66.2		44.1		37.3		29.3		11.2	
Situations of lifetime intimate partner violence		0.519		0.658		0.412		0.230		0.388
No (*n*, 177)	60.5		40.6		36.7		29.9		11.1	
1–3 (*n*, 62)	66.1		43.1		27.7		14.5		6.2	
4–6 (*n*, 5)	80.0		60.0		40.0		··		0.0	
Sex work (yes) (*n*, 33)	48.5	0.078	44.1	0.749	29.4	0.510	18.2	0.305	5.9	0.429
Private healthcare insurance (yes) (*n*, 154)	68.8^b^	0.006	39.4	0.341	34.4	0.968	34.4^b^	<0.001	10.6	0.463
Time of use of PrEP (months)		0.029		0.623		0.880		0.746		0.428
Up to 12 (*n*, 7)	28.6		28.6		28.6		14.3			
13–24 (*n*, 159)	59.1		43.5		35.4		26.4		11.2	
25–32 (*n*, 78)	71.8^b^		39.0		32.9		24.4		7.3	

^a^ All reported having sexual practices with men.

^b^ Standardized residual analysis ≥ 1.96 (Excess cases).

^c^ Standardized residual analysis ≤ 1.96 (Absence of cases).

An increased chance of choosing telehealth (Table 4) was associated with experience with PrEP and access to healthcare. Noteworthy, the clinical follow‐up in the southern region of the country, particularly in the cities of Curitiba and Porto Alegre (aOR: 14.48; 95% CI: 3.31–63.44; aOR: 3.16; 95% CI: 1.62–6.15, respectively); a more extended period of PrEP use (aOR from 25 to 32 months of use: 4.90; 95% CI: 1.32–18.25), not being on PrEP at the time of choice (aOR: 2.91; 95% CI: 1.40–6.06) and having private health insurance (aOR: 1.91; 95% CI: 1.24–2.94). The higher risk for HIV, notably sex without condoms in more than half of active anal intercourse with casual partners, reduced the chances of opting for telehealth (aOR: 0.40, 95% CI: 0.22–0.74). Sex work lost the effect of telehealth when variables related to experience with PrEP were included. No demographic indicators were associated with this choice.

**Table 4:** Adjust odds ratio and 95% confidence intervals for the choice of telehealth

		95% CI		
Characteristics	aOR	Lower limit	Upper Limit	*p*
**Vulnerability (last 6 months**)				
Sex work				
No	1			
Yes	0.74	0.42	1.29	0.291
**HIV risk (last 6 months)**				
Sexual partnership				
Steady and casual	1			
Only steady	1.13	0.46	2.73	0.795
Only casual	0.71	0.47	1.01	0.109
Active anal sex without a condom with a casual partner				
No	1			
Up to half of the relations	1.07	0.67	1.69	0.791
More than half of the relations	0.40	0.22	0.74	0.003
**Experience with PrEP and access to healthcare**				
Time of use of PrEP at the time of choice of follow‐up (months)				
Up to 12	1			
13–24	4.77	1.27	17.92	0.021
25–32	4.90	1.32	18.25	0.018
Being on PrEP at the time of the choice of follow‐up				
In use	1			
Discontinued	2.91	1.40	6.06	0.004
City of follow‐up PrEP				
São Paulo (testing centre)	1			
Curitiba (testing centre)	14.48	3.31	63.44	<0.001
Fortaleza (infectious diseases hospital)	1.21	0.59	2.48	0.608
Porto Alegre (HIV outpatient clinic)	3.16	1.62	6.15	0.001
Ribeirão Preto (HIV outpatient clinic)	1.21	0.49	3.02	0.684
Has private healthcare insurance				
No	1			
Yes	1.91	1.24	2.94	0.003

### 3.2 Discontinuation

Sixteen participants (3.4%) left the study because they moved to another city (6; 1.3%) or switched to an event‐driven regimen (10; 2.1%), with a similar distribution between the follow‐up types. In total, 110 participants (23.4%) discontinued PrEP: 53 telehealth and 57 in‐person. Of these, 30 (14 telehealth and 16 in‐person) were not followed up after choosing. Additionally, 20 participants (4.2%) changed the type of follow‐up, and most (14; 5.7% of telehealth) returned to in‐person follow‐up. The decision to return was mainly driven by personal and organizational challenges in conducting remote assessments and the physical requirement to visit the service for tests or medication pickups.

PrEP discontinuation curves showed a homogeneous pattern by follow‐up type (log‐rank 0.112; Tarone−Ware 0.113), with a mean time of PrEP use for post‐telehealth choice of 1.6 years (95% CI: 1.5–1.7; max 1.9 years) and 1.5 years for in‐person (95% CI: 1.4–1.6; max 1.9 years). The risk of discontinuing PrEP at 6, 12 and 18 months were similar, with 11.0% (95% CI: 7.7–14.3), 17.2% (95% CI: 13.8–20.6) and 26.0% (95% CI: 22.6–29.4) for telehealth and 14.0% (95% CI: 11.4–16.6), 21.4% (95% CI: 18.7–24.1) and 33.8% (95% CI: 29.1–38.5) for in‐person, respectively.

**Figure 1:**
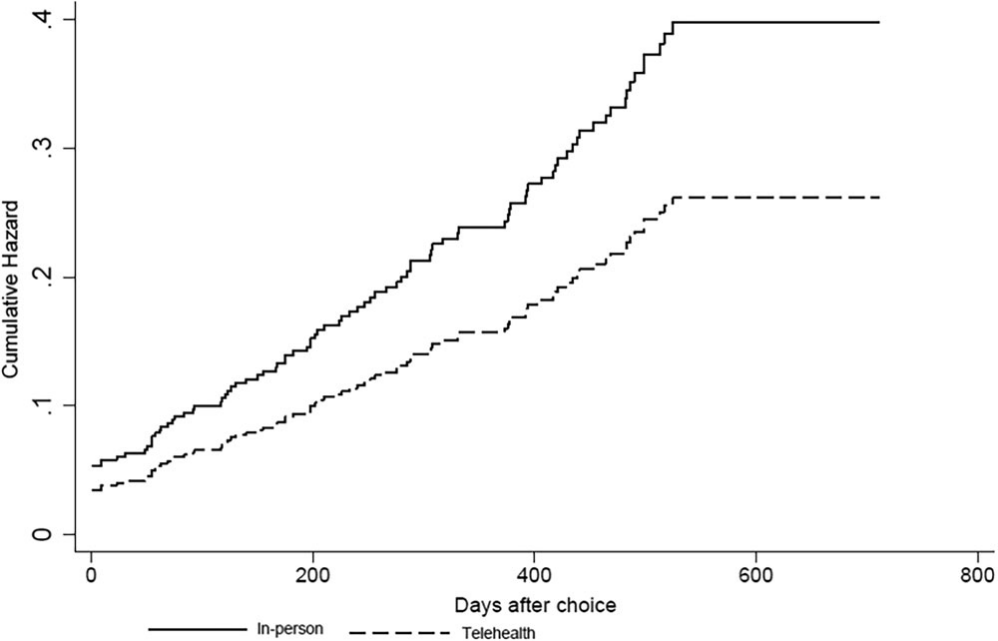
Risk function adjusted by the Cox model for discontinuation of PrEP after choosing the type of follow‐up.

When adjusted using the Cox model (Figure 1), the risk of discontinuing PrEP decreased by 34% (adjusted hazard ratio [aHR], 0.66; 95% CI: 0.45–0.97) in telehealth. The reduction was even more significant (Supplementary Material) when the participants were classified according to the last type of follow‐up performed in the study (aHR: 0.60; 95% CI: 0.41–0.89) and when excluded people without follow‐up after choice (aHR: 0.55; 95% CI: 0.35–0.87). After adjustment, the increased risk of discontinuation (Supplementary Material) was associated with not being on PrEP at the time of the choice (aHR: 4.21, 95% CI: 2.61–6.79) and not having used condoms in more than half of them during anal intercourse (aHR: 1.65; 95% CI: 1.03–2.64).

### 3.3 Adherence

**Figure 2:**
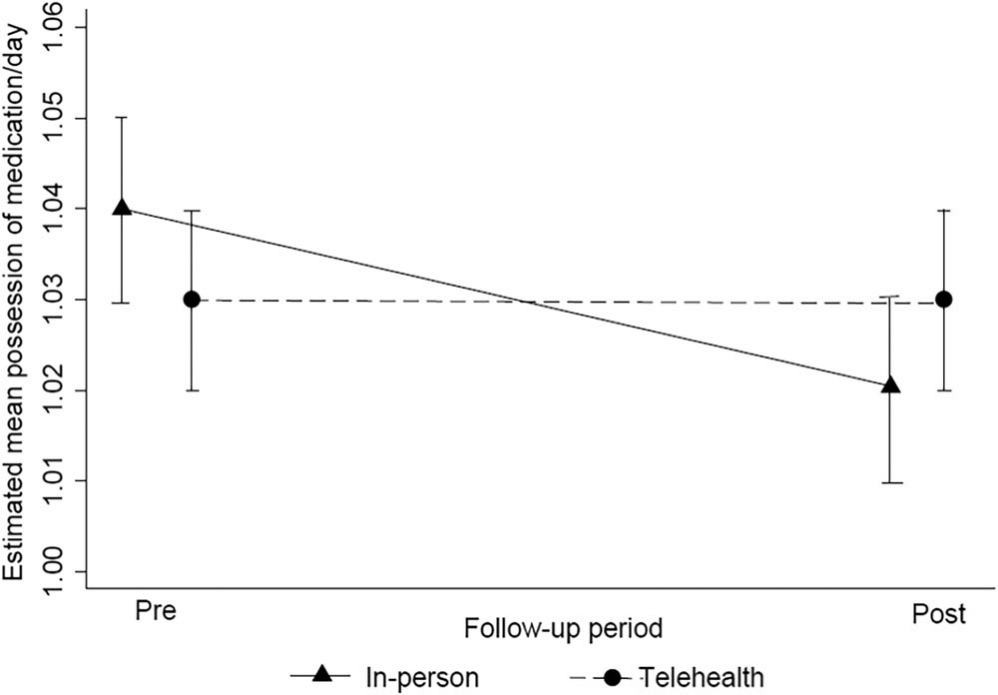
Estimation of the mean rate of medication possession in the pre‐ and post‐choice periods of the type of clinical follow‐up. *Notes*: 1. Performed using a mixed linear model 2. Descriptive level of the mixed linear Model (p): in‐person and telehealth follow‐up (0.486); pre‐ and post‐choice time (0.054); Interaction type of follow‐up and time (0.245) 3. Number of observations = 5819, relative to 470 individuals

Medication was withdrawn twice or more from 401 participants. The mean MPR values (Figure 2) were similar for telehealth and in‐person care (*p* = 0.486) and between the pre‐ and post‐onset periods (*p* = 0.054), with mean MPR indices higher than 1 medication/day. A slight decline in MPR (−0.01; 95% CI: −0.03 to 0.01) occurred in person between the pre‐ and post‐choice and was unrelated to the type of follow‐up or time (follow‐up/time interaction, *p* = 0.245).

No significant differences (*p* = 0.259) were found between the groups with MPR <1/day and MPR ≥ 1/day. The proportion of people with MPR <1/day was 19.5% (95% CI: 14.2–24.9) in telehealth and 24.2% (95% CI: 18.0–30.4) in in‐person. This remained unchanged (Table 5) after the adjustment (*p* = 0.921). The only variable associated with an MPR <1/day was the duration of PrEP use, and an increase of 1 day of use reduced the chance of insufficient adherence by 2% (aOR, 0.998; 95% CI: 0.997–0.999).

**Table 5:** Adjust odds ratios (aORs) and 95% confidence intervals (95% CI) for adherence to PrEP lower than the possession of 1 medication/day

Characteristics	aOR	95% CI		*p*
		Lower limit	Upper Limit	
Follow‐up chosen				
Telehealth	1			
In‐person	1.02	0.67	1.57	0.921
Age (years)	0.98	0.95	1.00	0.070
Passive anal sex without a condom with a casual partner in the last 6 months				
No	1			
Up to half of the relationships	0.72	0.43	1.22	0.225
More than half of the relationships	1.05	0.58	1.91	0.874
Time of use of PrEP (days)	0.998	0.997	0.999	<0.001
Type of partnership in the last 6 months				
Only fixed	1			
Only casual	1.18	0.51	2.73	0.691
Fixed and casual	1.20	0.53	2.68	0.663
Follow‐up situation at the time of choice				
In use	1			
Discontinued	1.02	0.46	2.26	0.952

### 3.4 STIs and HIV

**Figure 3:**
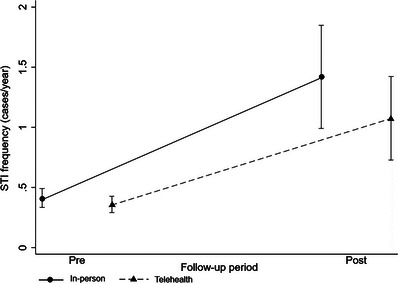
Estimates of the frequency of STIs (cases/year) by type of chosen follow‐up and for the pre‐ and post‐choice periods. *Notes*: 1. Performed using generalised estimation equations with negative binomial distribution. 2. Multiple comparisons between Bonferroni correction follow‐ups – Pre (p = 0,772) and Post (p = 0.438). 3. Multiple comparisons between times with Bonferroni correction telehealth (p < 0.001) and in‐person (p < 0.001). 4. Adjustment variables: age, education, partnership, prostitution, active anal sex without condom with casual partner, passive anal sex without condom with casual partner and total number of partners.

A total of 300 syndromes indicative of STIs were recorded in telehealth and 420 in‐person, with an increase (*p* <0.001) in the mean frequency of occurrence between the pre‐ and post‐choice periods (Figure 3), both in telehealth (0.359–1.075 cases/year) and in in‐person (0.405–1.420 cases/year). The intensity of the increase did not differ between follow‐up types (follow‐up/time interaction *p* = 0.528), showing that the pattern of average occurrence of STIs in telehealth and in‐person was similar in the pre‐ (*p* = 0.772) and post‐onset periods (*p* = 0.438).

No HIV infections occurred.

## 4 DISCUSSION

We evaluated the telehealth model using asynchronous evaluations over an average of 1.6 years. Acceptability of telehealth was associated with more experience with PrEP and a lower HIV risk, possibly indicating a profile of clients that represents a lower demand for healthcare services. Furthermore, telehealth has improved the effectiveness of PrEP by reducing discontinuity by 1/3 without compromising adherence, STI diagnosis and the risk of acquiring HIV.

These results should be interpreted considering that a part of the study was conducted during the COVID‐19 pandemic. This might have influenced telehealth's acceptability or been an additional barrier to in‐person follow‐ups. Additionally, healthcare professionals may engage in the delivery of telehealth services differently. Furthermore, confounding factors might not have been sufficiently controlled for, especially those related to the daily lives of individuals, such as the type of work, conceptions of self‐care and the connection to services. This may explain the differences in acceptance per city and follow‐up services. We tried to minimize these effects by developing a telehealth protocol with the participation of services and clients. We used different analytical strategies to compare telehealth and in‐person follow‐up.

Nonetheless, noteworthy, a 1/3 reduction existed in the risk of discontinuing PrEP in telehealth, which was greater among individuals who opted for initiation and remained on telehealth. The magnitude of this risk reduction has not yet been reported, although an association between PrEP persistence and telehealth is known [23, 24].

This result may be related to the ability of telehealth to help overcome barriers to PrEP retention, such as work routines, fear of stigma, distance from services and waiting time for consultations [25, 26]. Notably, most of those who chose telehealth mentioned that PrEP would not require going to services or that telehealth helped them avoid conflicts with personal commitments. Furthermore, when these assumptions were challenged, individuals discontinued telehealth and resumed in‐person follow‐up. Thus, the efficacy of telehealth may be attributed to the simplification of clinical follow‐up and the process of “demedicalizing” PrEP, which establishes a closer connection to the daily lives of individuals.

The results also showed the potential of telehealth to decongest services, removing people with access to private health, a lower risk of HIV and a better ability to manage PrEP use from in‐person care. This allows for better rationalization of the service and, consequently, expansion of coverage [24, 27] while prioritizing in‐person care for those who need it most. This includes intercurrence consultations for individuals who are under telehealth follow‐up. This demand accounted for approximately 5% of the consultations in remote follow‐up, mainly due to changes in the exams, suspected STIs or reintroduction of PrEP. This decongestion of services may be more relevant in low‐ and middle‐income countries, as the health services tend to operate at the capacity limit, resulting in more relevant access barriers. However, it should be emphasized that our experience in implementing telehealth involved efforts to reorganize work processes, incorporate new technologies, adopt remote management procedures, engage clients and train professionals. Noteworthy, in this process, some professionals expressed concerns about losing control over the users’ care strategies during the implementation process of remote follow‐up. Transposing this vision was essential for the proper development of the protocol.

One frequent concern regarding telehealth is the possibility of negatively affecting adherence to PrEP or STI diagnoses. Our results indicate another direction, consolidating the evidence [9, 12, 15] that telehealth allows the maintenance of a standard of care equivalent to in‐person care. This may have resulted from the study protocol, which offered telehealth to those on PrEP for a minimum period. Additionally, forms for self‐reporting of signs and symptoms, maintenance of screening tests and the stimulus to seek services in case of suspicion allowed the identification of STIs to follow a similar pattern between telehealth and in‐person. This is a relevant result because a technology that negatively interferes with the relationship between PrEP and STIs is less acceptable.

Regarding the limitations of the telehealth protocol, individuals with a history of discontinuation mainly chose telehealth and subsequent discontinuation. It is possible to assess whether a telehealth intervention before the 6 months of PrEP adoption, as implemented in this study, would significantly impact avoiding discontinuity by reducing barriers promptly for these individuals. Furthermore, individuals with a low adherence/continuity history are more vulnerable to HIV [27]. Therefore, various PrEP delivery strategies [28] (e.g. community mobile clinics, pharmacy‐led PrEP and telehealth) or regimens (event‐driven and long‐term) must be combined to effectively address their needs.

## 5 CONCLUSIONS

Telehealth can strategically scale PrEP by increasing PrEP persistence without changing adherence or the ability to diagnose STIs. It can also contribute to a more significant rationalization of services, allowing the prioritization of in‐person care for people at a greater risk of HIV and social vulnerability. Evaluation of a model with asynchronous evaluation increases the options for PrEP programmes closer to individuals’ needs.

## COMPETING INTERESTS

All authors declare that they have no competing interests.

## AUTHORS’ CONTRIBUTIONS

AG and LAS contributed to conceptualization, data curation, formal analysis, funding acquisition, writing original draft and review final version; DLE, RM, EA, RAM, LQW, LASN, JCVS and MK with project administration, data curation, supervision, writing original draft and review final version; MAHK with the formal analysis and review final version; EMZ, AFL, MFTP, MTC and JEN with initial draft and review of final version; and MME with conceptualization, formal analysis, writing original draft and review the final version.

## FUNDING

This study was funded by the Brazilian Ministry of Health and Pan American Health Organisation (grant number: SCON2019‐00356).

## COMBINE! RESEARCH GROUP

Alan Clausen da Silveira, Bruno Silva Kauss, Dennis Soares Leite, Jonatan da Rosa Pereira Da Silva, Maria Eugênia Paiva do Amaral, Romário Miranda Alexandre, Thiago Souza Reis, Vanessa Souza Brito and Wilver Cunha Portella.

## Supporting information


**Table S1**. Adjust hazard ratio (aHR) and 95% confidence intervals (95% CI) for discontinuation of PrEP, according to the first type of follow‐up choice in the study
**Table S2**. Adjust hazard ratio (aHR) and 95% confidence intervals (95% CI) for discontinuation of PrEP, according to the last type of follow‐up performed in the study
**Table S3**. Adjust hazard ratio (aHR) and 95% confidence intervals (95% CI) for discontinuation of PrEP, according to the last type of follow‐up performed and excluding people without follow‐up after the first choice in the study

Supplementary File: TelePrEP Platform Development History.
Screenshot 01: Login screen.
Screenshot 02: Professional profile initial menu.
Screenshot 03: Screen for scheduling face‐to‐face appointments.
Screenshot 04: Access screen to the remote PrEP clinical evaluation form.
Screenshot 05: Tab for filling in the results of routine clinical exams.
Screenshot 06: Tab for final conduct and PrEP prescription.
Screenshot 07: Chat screen between professional and user.

## Data Availability

Individualized and anonymized data can be accessed by researchers upon presentation of projects and ethical approval.
