# Supplementary material for: Telehealth effectiveness for pre‐exposure prophylaxis delivery in Brazilian public services: the Combine! Study
Source: J Int AIDS Soc. 2023 Sep 27;26(9):e26173. doi: 10.1002/jia2.26173 (PMC10534058; doi:10.1002/jia2.26173)
Supplement: Supplementary file 2 — Supplementary File: TelePrEP Platform Development History. Screenshot 01: Login screen. Screenshot 02: Professional profile initial menu. Screenshot 03: Screen for scheduling face‐to‐face appointments. Screenshot 04: Access screen to the remote PrEP clinical evaluation form. Screenshot 05: Tab for filling in the results of routine clinical exams. Screenshot 06: Tab for final conduct and PrEP prescription. Screenshot 07: Chat screen between professional and user. [file JIA2-26-e26173-s002.docx]

**Supplementary File: TelePrEP Platform Development History.**

The telePrEP clinical management platform was developed in connection with the medication logistic control system (SICLOM in Portuguese) of the Brazilian Ministry of Health to allow interaction between face-to-face and telehealth services. Thus, information from clinical follow-up forms, test results and prescription history was automatically transferred between the telePrEP platform and the national system that records PrEP dispensing information.

The development of the web platform involved conducting interviews with professionals and managers from each of the services that were part of the study. The aim was to understand better their work processes, their thoughts on remote PrEP consultations, and the operational and technical requirements for implementing this method of clinical follow-up. The interviews also focused on identifying appropriate measures for virtual interaction between the service and the participants and strategies to manage absenteeism and low adherence to PrEP.

Each service's unique needs were taken into consideration during this process. For the development of the clinical evaluation forms, meetings were held with medical and psychosocial counselling professionals to understand the aspects considered as a warning to start/deepen an investigation or define a clinical approach in PrEP follow-up. PrEP users were also interviewed to understand their perceptions of the questions included in the original SICLOM forms and problems that could lead to incorrect filling out to inform the development of the telePrEP platform. In addition, we identified the need to include questions that could highlight the need for consultations for counselling and/or support to deal with situations associated with more significant impairment of the excellent use of PrEP and mental health, such as the occurrence of cases of violence or stigma.

Given this, three access modules to the platform were developed, one for the unit's management team, one for PrEP users and another for health professionals. The platform was developed using the PHP programming language (a recursive acronym for PHP: Hypertext Preprocessor ) and the Symfony framework (a free fullstack framework for developing web applications using the PHP language and the MVC paradigm). Mysql database for storing all the information filled in on the platform. All these tools run on the Linux platform and use the HTTP NGINX webserver to access participants via the web or any device with internet access.

Below, we present the main access screens for users and professionals.

**Screenshot 01: Login screen.**


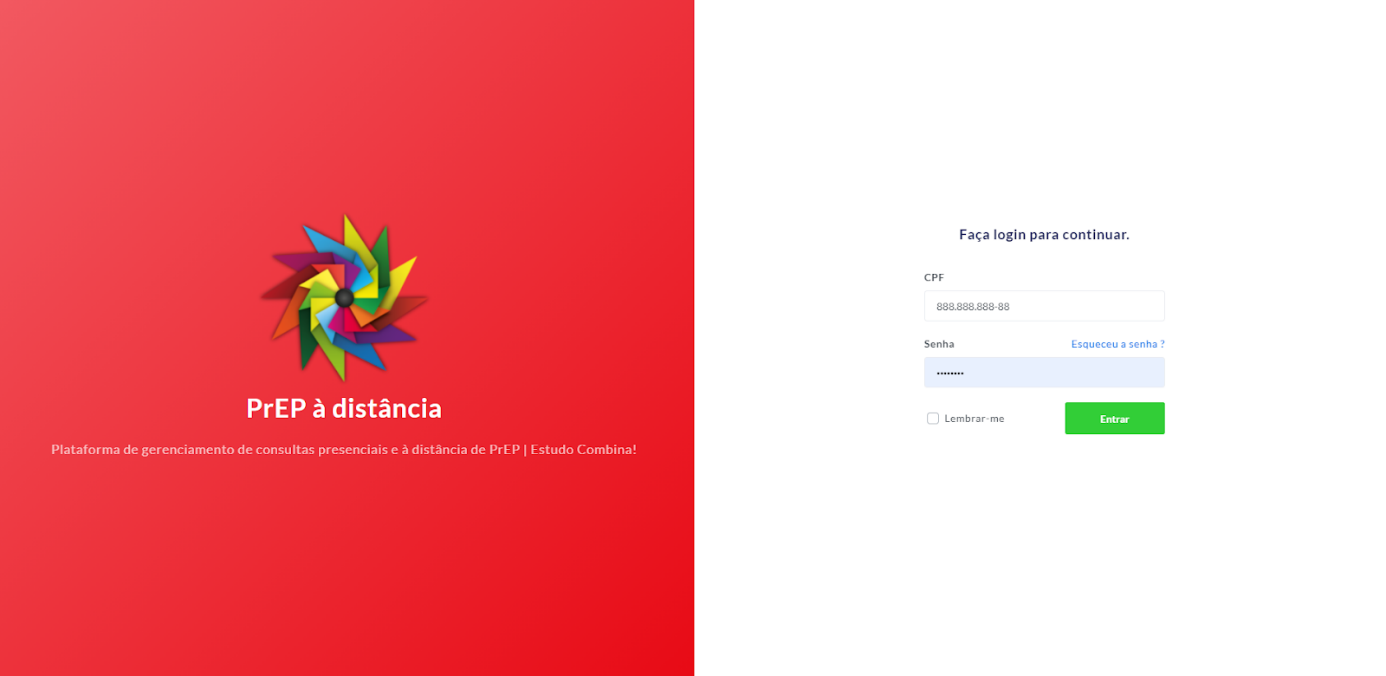


*Description: This screen is identical to the user's profile.*

**Screenshot 02: Professional profile initial menu.**


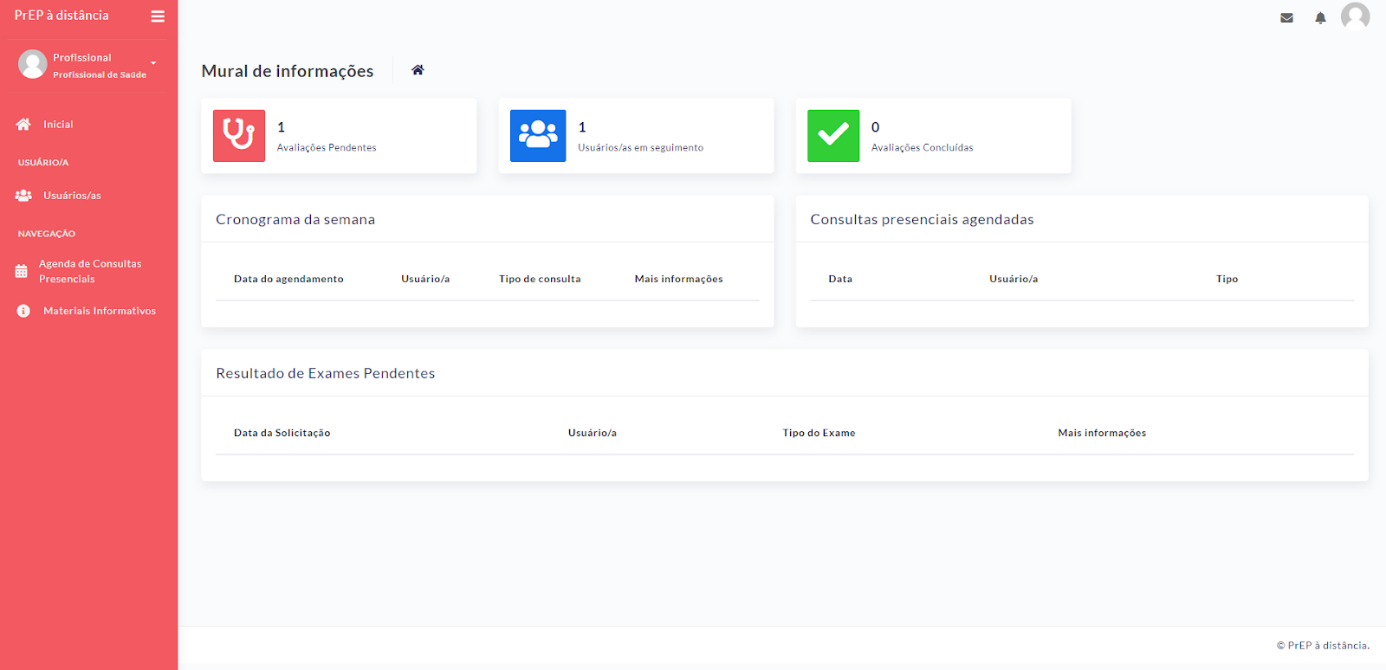


*Description: On the side tab (highlighted in red on the left), buttons direct you to the individual profile of the professional where you can change registration information and password, search button and access personal user profiles, a button for scheduling appointments face-to-face sessions and link to complementary information materials. On the right, an information wall with a summary of the main information, such as the schedule of evaluations planned for the week, in-person consultations scheduled for the week, and test results pending completion on the platform, among other information.*

**Screenshot 03: Screen for scheduling face-to-face appointments.**


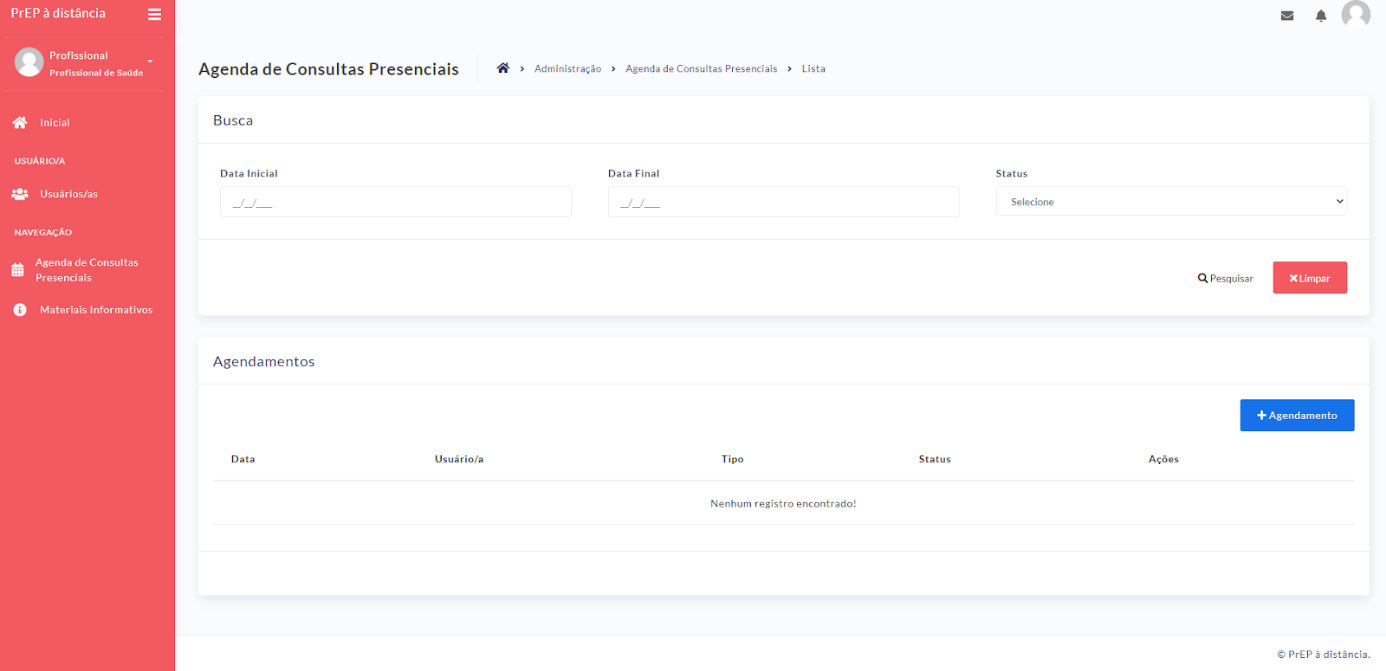


**Screenshot 04: Access screen to the remote PrEP clinical evaluation form.**


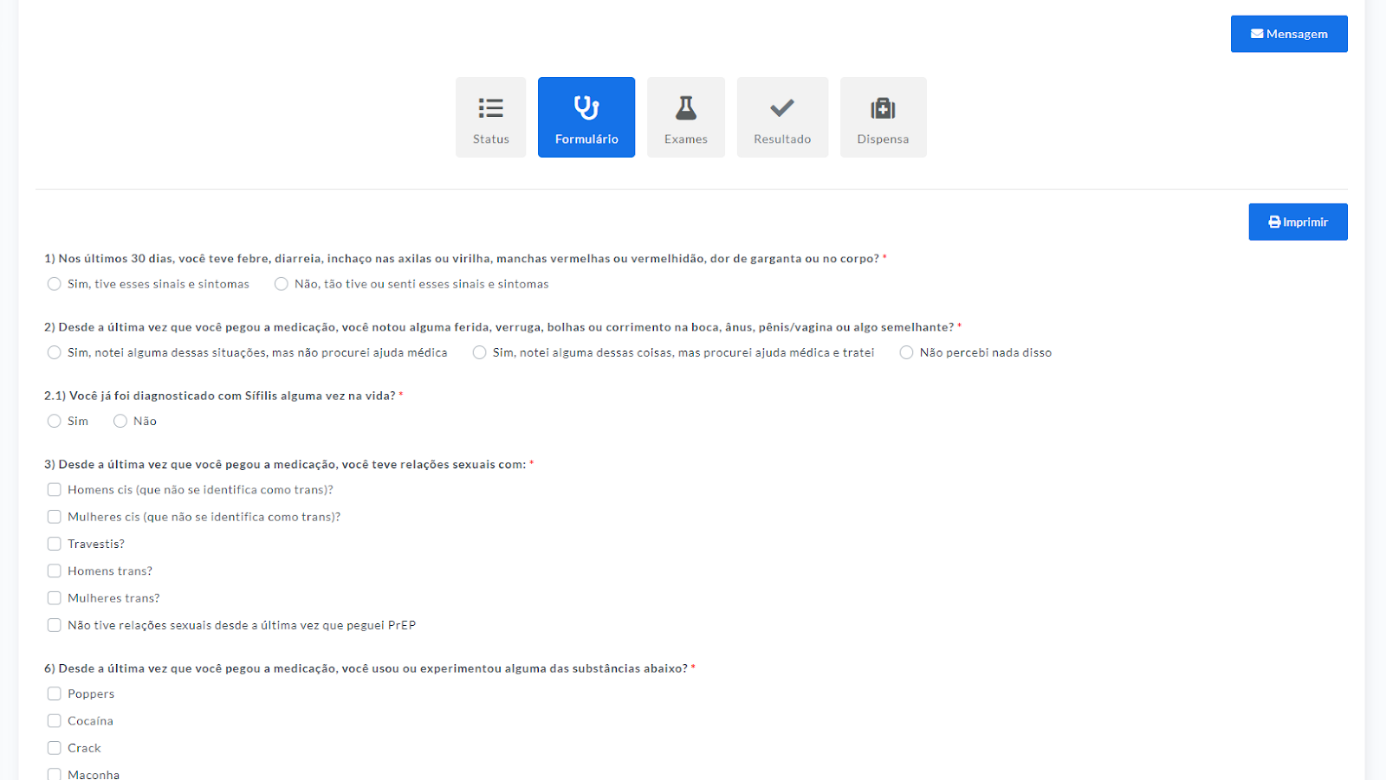


**Screenshot 05: Tab for filling in the results of routine clinical exams.**


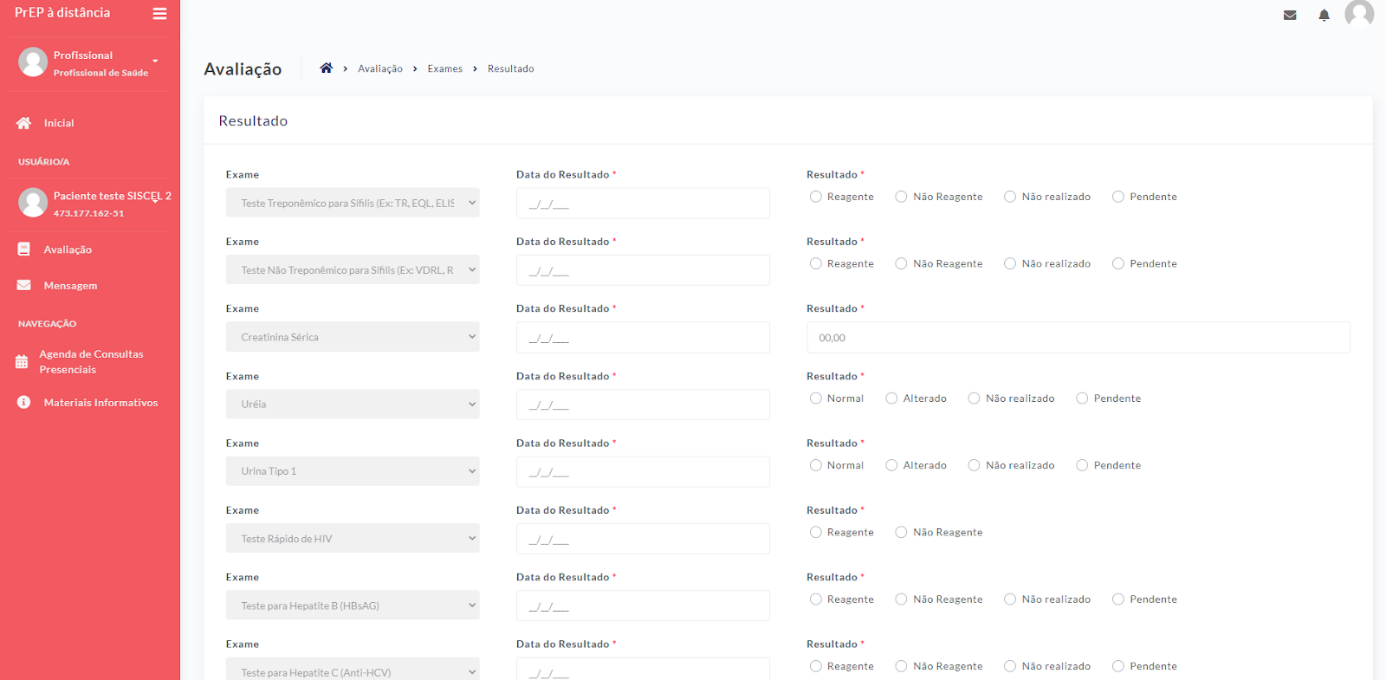


**Screenshot 06: Tab for final conduct and PrEP prescription.**


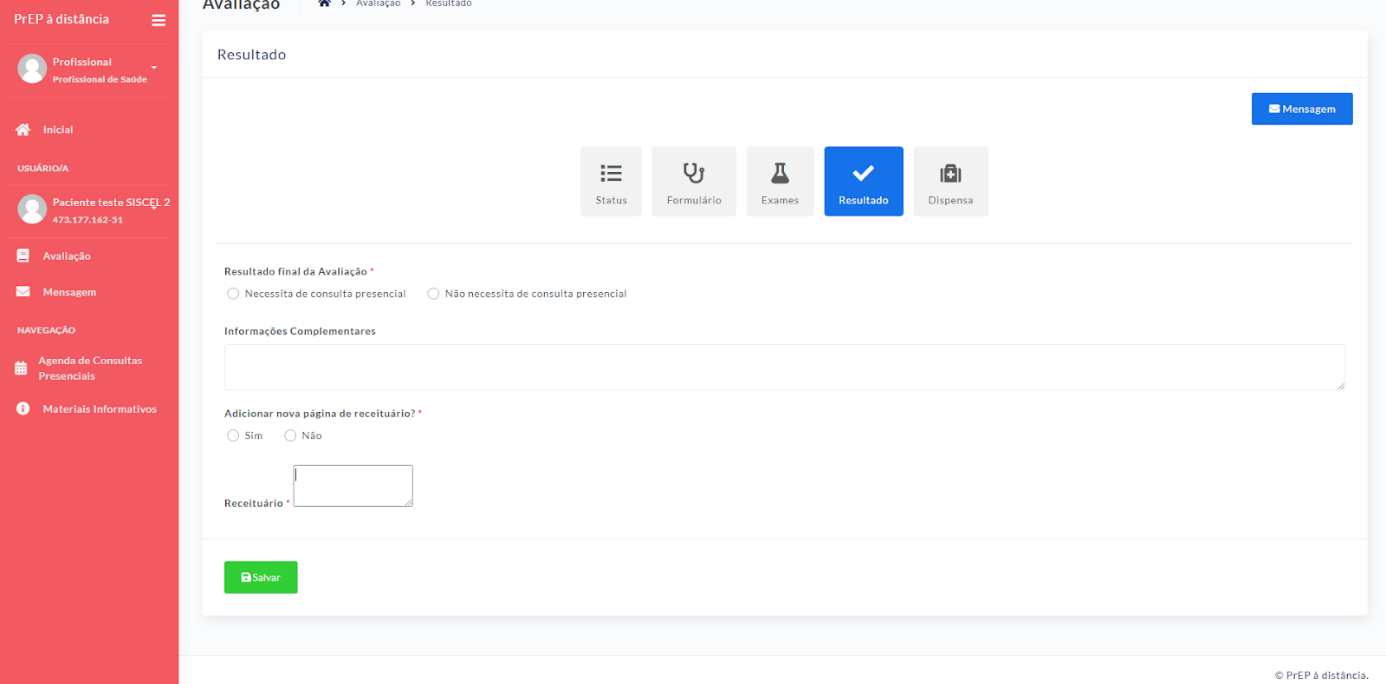


**Screenshot 07: Chat screen between professional and user.**


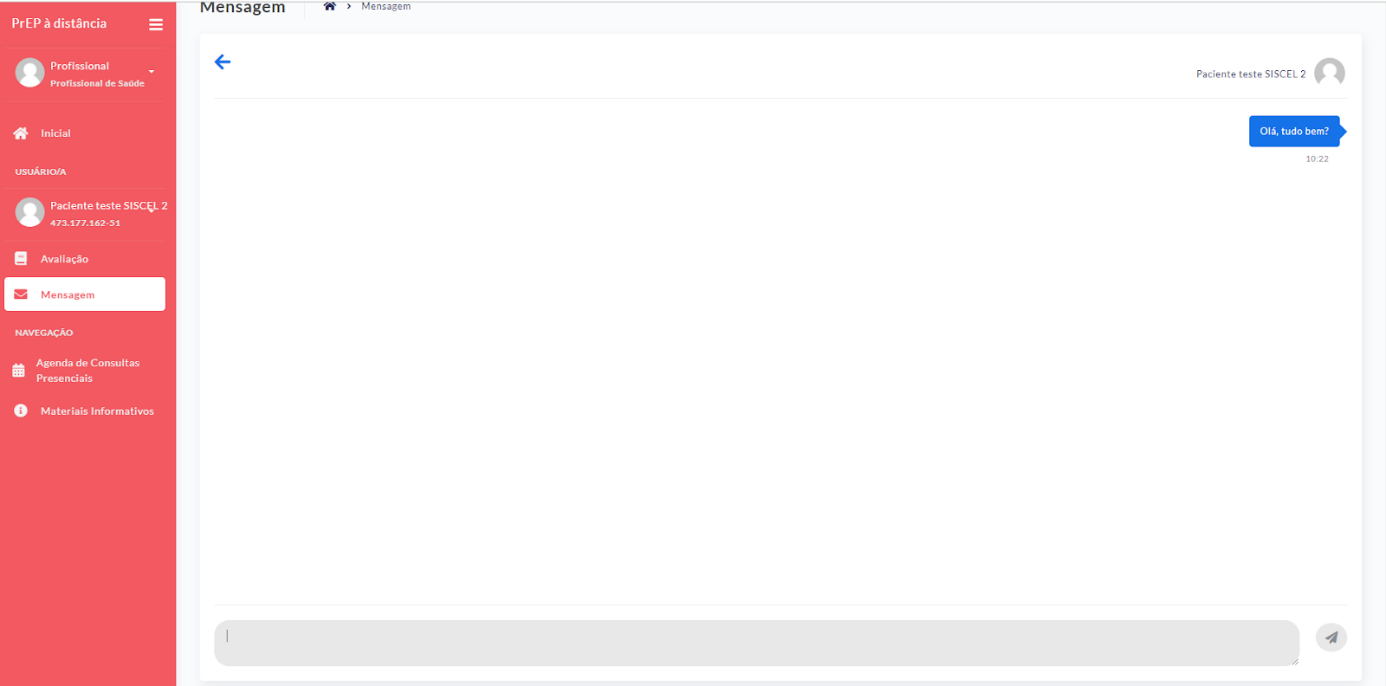


*Description: Alert emails are sent to the professional and user, informing them when there is a new unread message.*
